# Involvement of integrin αvβ3 in thyroid hormone-induced dendritogenesis

**DOI:** 10.3389/fendo.2022.938596

**Published:** 2022-08-22

**Authors:** Winda Ariyani, Wataru Miyazaki, Izuki Amano, Noriyuki Koibuchi

**Affiliations:** ^1^ International Research Fellow of Japan Society for the Promotion of Science, Tokyo, Japan; ^2^ Department of Integrative Physiology, Gunma University Graduate School of Medicine, Maebashi, Gunma, Japan; ^3^ Department of Bioscience and Laboratory Medicine, Hirosaki University Graduate School of Health Science, Hirosaki, Aomori, Japan

**Keywords:** T_3_, T_4_, rT_3_, neuron, astrocyte, F-actin, development, cerebellum

## Abstract

Activation and/or modulation of the membrane-associated receptors plays a critical role in brain development. Thyroid hormone (TH) acts on both nuclear receptors (thyroid hormone receptor, TR) and membrane-associated receptors, particularly integrin αvβ3 in neurons and glia. Integrin αvβ3-mediated signal transduction mediates various cellular events during development including morphogenesis, migration, synaptogenesis, and intracellular metabolism. However, the involvement of integrin αvβ3-mediated TH action during brain development remains poorly understood. Thus, we examined the integrin αvβ3-mediated effects of TH (T_3_, T_4_, and rT_3_) in the neurons and astrocytes using primary cerebellar culture, astrocyte-enriched culture, Neuro-2A clonal cells, and co-culture of neurons and astrocytes. We found that TH augments dendrite arborization of cerebellar Purkinje cells. This augmentation was suppressed by knockdown of integrin αvβ3, as well as TRα and TRβ. A selective integrin αvβ3 antagonist, LM609, was also found to suppress TH-induced arborization. However, whether this effect was a direct action of TH on Purkinje cells or due to indirect actions of other cells subset such as astrocytes was not clarified. To further study neuron-specific molecular mechanisms, we used Neuro-2A clonal cells and found TH also induces neurite growth. TH-induced neurite growth was reduced by co-exposure with LM609 or knockdown of TRα, but not TRβ. Moreover, co-culture of Neuro-2A and astrocytes also increased TH-induced neurite growth, indicating astrocytes may be involved in neuritogenesis. TH increased the localization of synapsin-1 and F-actin in filopodia tips. TH exposure also increased phosphorylation of FAK, Akt, and ERK1/2. Phosphorylation was suppressed by co-exposure with LM609 and TRα knockdown. These results indicate that TRs and integrin αvβ3 play essential roles in TH-induced dendritogenesis and neuritogenesis. Furthermore, astrocytes-neuron communication *via* TR-dependent and TR-independent signaling through membrane receptors and F-actin are required for TH-induced neuritogenesis.

## Introduction

Thyroid hormones (THs), in the form of thyroxine (T_4_) or 3,5,3’-tri-iodo-L-thyronine (T_3_), are essential for developmental and metabolic processes, including brain development (1–3). TH regulates various developmental processes including neuronal and glial proliferation, neuronal differentiation and migration, synaptogenesis, and myelination (3–5). Among TH target brain regions, the cerebellum plays an essential role in integrating and processing motor and sensory information to regulate balance, posture, precise movements, and motor learning (6–8). In addition, cerebellum also contributes to cognitive functions such as attention, emotion, working memory, language, and visuospatial navigation (9). The cerebellum consists of various cell types, including Purkinje cells, granule cells, basket cells, stellate cells, and astrocytes, and is comprised of a cytoarchitecture array of stripes and zones (8). Perinatal hypothyroidism is reported to affect neurogenesis and differentiation in the cerebellar cortex (4, 10). Therefore, the developing cerebellum may represent an excellent model for studying the effects of TH on brain development. Despite the detailed description of impaired cerebellar development caused by hypothyroidism, the molecular mechanisms underlying TH-induced cellular morphogenesis remain unclear.

The activity of TH in the brain is regulated in part by deiodination, a process that activates or inactivates TH (3, 11). The predominant deiodinases expressed in the brain are type 2 and 3 iodothyronine deiodinases (D2 and D3, respectively), which are products of the DIO2 and DIO3 genes, respectively. DIO2 and DIO3 have variable expression between cell types. DIO2 is expressed by astrocytes where it converts T_4_ to T_3_, an active form of TH, to regulate cellular functions of nearby cells. DIO3 is expressed by neurons where it degrades T_3_ and T_4_ to rT_3_ (3,3’,5’-triiodothyronine) and T_2_ (3,3’-diiodothyronine), respectively (3, 12). Although T_3_ and T_4_ are the predominant TH in the brain, several studies have found that rT_3_ also has physiological functions in both neurons and glia. rT_3_ is involved in Dio2 turnover and regulates the actin cytoskeleton to control neurite outgrowth and glial migration (1, 3, 11–13). Thus, study of the physiological roles of individual TH derivatives are required to fully understand the effect of TH signaling on brain development.

TH is known to act by binding to the nuclear TH receptor (TR), which has two major isoforms, TRα and TRβ, leading to activation of various signaling pathways (1, 14, 15). A TH signalling pathway classification has recently been described (15). Type 1 represents TR-dependent signaling caused by direct binding to a DNA sequence termed the TH response element as homodimers or heterodimers with the retinoid X receptor (RXR). Type 2 is TR-dependent signaling of TH with indirect binding to DNA such as through AP1 (jun/fos) complexes to induce chromatin remodeling. Type 3 represents TR-dependent signaling of TH without DNA binding, in which TH binds with cytoplasmic TR to interact with kinases without binding to DNA. Type 4 is TR-independent TH signaling in which TH acts without binding to TR but through membrane receptors such as integrin αvβ3. The type 4 pathway is also termed non-genomic action of TH (15, 16). This wide variety of pathways may interact with each other to regulate cellular functions. Thus, studying the type 1 pathway alone may not be sufficient to fully understand the mechanisms underlying the activities of TH in brain development.

TR-independent TH signaling/non-genomic activity involves TH binding to the integrin αvβ3, leading to intracellular trafficking of specific proteins to the nucleus. Serine phosphorylation of trafficked proteins allows regulation of the transcription of specific target genes (13). Integrin controls various cellular events including cell migration, cortical layer formation, axon regeneration, and neurite outgrowth (17, 18). Upon activation, integrin activates FAK signaling through p190 RhoGEF, which controls axonal branching and synapse formation in the neurons (19). In addition, FAK also colocalizes with microtubules in a branched fork-like form to enhance neuronal cell migration (19, 20). These results indicate that TH binding to integrin αvβ3 may enhance neuritogenesis through various signaling pathways.

A recent study reported TH binding to integrin αvβ3 activates several signaling pathways that modulate cell proliferation, angiogenesis, and migration of tumor cells and specific non-malignant cells (16). In addition, integrin αvβ3 is highly expressed in the brain, and deletion of integrin αv or β3 during brain development in both glia and/or neurons leads to cerebral hemorrhage, aberrant dendritogenesis, and synaptogenesis leading to neurological impairments (21–23). Despite this evidence, it is currently unknown whether integrin αvβ3 activation by TH affects brain development, particularly in the cerebellum. Therefore, we aimed to determine the effects of integrin αvβ3 activation by TH during neuritogenesis using primary cerebellar culture and neuronal-derived clonal cell line, Neuro-2A. We also examined the downstream pathway of integrin αvβ3 activation in response to TH on protein phosphorylation and F-actin rearrangement. TH exposure affects neuritogenesis *via* both TRα-dependent and TR-independent (integrin αvβ3) signaling pathways. TH exposure also promotes the phosphorylation of FAK, Akt, and ERK1/2 and increases colocalize of F-actin and synapsin-1.

## Material and methods

### Chemicals

T_3_, T_4_, and rT_3_, were purchased from Sigma (St. Louis, MO, USA) with the purity >98%. Monoclonal antibodies against integrin αvβ3, clone LM609, as allosteric inhibitor of integrin αvβ3 was purchased from Millipore (Millipore Corporation, Temecula, CA, USA).

### Primary culture of mouse cerebellar cells

All animal experimentation protocols used in this study were ethically reviewed and approved by the Animal Care and Experimentation Committee, Gunma University (19-024, 17 December 2018). All experiments performed in this study follow the approved guidelines and regulations and made to minimize animal suffering and the number of animals used.

Primary culture of cerebellar cells was prepared as previously described (24, 25) with slight modifications. The C57BL/6 pregnant mice were purchased from Japan SLC (Hamamatsu, Japan). At birth, pups were euthanized under isoflurane anesthesia. Cerebellum were dissected then digested with papain dilution buffer, which is 0.1 M of phosphate-buffered saline (PBS) containing 0.2 unit/mL papain (Worthington, Lakewood, NJ, USA) 0.02 mg/mL DNase I (Sigma), 0.2 mg/mL l-cysteine, 5 mg/mL glucose (Wako, Japan), and 0.2 mg/mL bovine serum albumin (Intergen Company, Purchase, NY, USA). Cerebellar tissue was incubating at 36.5°C for 25 min with continuous shaking. Cells were resuspended in a cerebellar culture medium (Dulbecco’s modified Eagle’s medium, DMEM/F12 (Wako) containing 1% penicillin–streptomycin (Wako), 3.9 mM glutamine (Wako), 2.1 mg/ml glucose (Wako), 30 nM sodium selenite (Sigma), 20 mg/ml insulin (Sigma), and 200 mg/ml transferrin (Sigma)). Resulting suspensions of dissociated cells were counted and total of 3 × 10^5^ cells/0.3 mL cerebellar cells were plated in the poly-l-lysine coated 8 mm diameter wells of chamber slides (Lab-Tek; Nunc International, Rochester, NY, USA). T_3_, T_4_, or rT_3_ (Sigma) at concentration of 10 nM was added to the culture medium at 16–24 h after plating. Every three days, one-half of the medium was replaced with fresh medium and incubate in a 5% CO_2_ incubator at 37°C.

At 17 days after cultured, mixed cerebellar cells were fixed with 4% PFA. Cells were blocked with 5% fetal bovine serum (FBS) in PBS. Next, cerebellar cells were immunostained for Purkinje cells marker, which is mouse monoclonal anti-calbindin-D-28K antibody (1:200, Sigma), continued with secondary antibody, donkey anti-mouse IgG (H + L) Alexa Fluor^®^ 488 conjugate (1:200) (Thermo Fisher Scientific, Inc, Waltham, MA, USA). Cell nuclei were stained with DAPI. Purkinje cells were examined under a laser confocal scanning microscope (Zeiss LSM 880, Carl Zeiss Microscopy GmbH, Jena, Germany). For each experiment, 20–50 Purkinje cells were randomly selected to quantify dendrite arborization and perform Sholl analyses. Total area of dendrite arborization was measured by tracing the outline of cells and dendritic branches using Fiji ImageJ software (NIH, Bethesda, MD, USA). Sholl analysis was performed by manually tracing each Purkinje cell using Adobe illustrator (Adobe Inc., San Jose, CA, USA). The overlaid of transparent layer of the Purkinje cells was used to trace the Purkinje cells, followed by Sholl analysis using Fiji ImageJ software (NIH). Intersections between dendrites and each concentric circle were measured and analyzed. Data are showed as the mean ± standard error of the mean (SEM). More than three independent experiments were performed. Results were consistent between each experiment. A representative result from one experiment is shown.

### Primary culture of mouse cerebellar astrocytes

Primary culture of mouse cerebellar astrocytes (25, 26) was prepared using C57BL/6 pregnant mice (Japan SLC). Cerebella from postnatal day one of pups were dissected then digested in Hank’s balanced salt solution (Wako) containing 2.5% trypsin (Wako). Cerebellar tissue was incubated at 37°C for 30 min with continuous shaking. Mixed cerebellar tissue was centrifuge at 3500 rpm for 15 minutes at 4°C, the supernatant was discarded, and cells were resuspended in an astrocyte culture medium (high-glucose DMEM (Wako), 10% heat-inactivated FBS, and 1% penicillin/streptomycin). Cells were counted and 10–15 × 10^6^ cells were plated on Collagen I coated 10–cm dishes (Iwaki, Japan), then incubated at 37°C in a CO_2_ incubator. The astrocyte culture medium was replaced with PBS on day 3 *in vitro* (DIV3). Oligodendrocyte precursor cells were removed by shaken the dishes for 2–5 minutes. Discard the supernatant then replaced with a fresh astrocyte culture medium. On DIV7, astrocytes were harvested and then plated on Collagen I coated 6 or 24 well dishes (Iwaki). Cells were then used for co-culture studies.

### Mouse Neuro-2A cells and co-culture studies

Neuro-2A cells, mouse neuroblastoma-derived clonal cells, were obtained from the Japanese Cancer Research Resources Bank cell bank (National Institutes of Biomedical Innovation, Health, and Nutrition, Tokyo, Japan). Neuro-2A cells were cultured in DMEM (Wako) supplemented with 10% FBS and 100 U/mL penicillin and 100 µg/mL streptomycin (Wako) with 5% CO_2_ at 37°C. Powdered charcoal and AGXI-8 resin (Bio-Rad, Hercules, CA, USA) were used to stripped the serum from hormones by constantly mixing and ultrafiltration. Neuro-2A cells were counted, then 1 × 10^5^ cells/mL per well were plated in poly-l-lysine coated 6- or 24-well plates the in DMEM + 10% FBS. Culture medium was changed to DMEM+1% FBS on the next day with or without 10 nM of T_3_, T_4_, or rT_3_ to trigger differentiation (25). Cells then incubated at 37°C in a CO_2_ incubator for one to three days, then used for RT-qPCR and immunocytochemistry. Neuro-2a cells were fixed with 4% PFA, followed by blocked with 5% FBS in PBS for 30 minutes. Cells were then immunostained with neuronal marker, mouse anti-β-tubulin III (1:200) and rabbit anti-doublecortin (C–terminal) (1:200) (Sigma) antibodies, continued with secondary antibody by donkey anti-mouse Alexa Fluor^®^ 594 (1:200) and donkey anti-rabbit Alexa Fluor^®^ 488 conjugate (1:200) (Thermo Fisher Scientific, Inc.). DAPI was used to stained the cell nuclei. Neuro-2A cells were observed under a laser confocal scanning microscope (Zeiss LSM 880, Carl Zeiss Microscopy GmbH). ImageJ Fiji (NIH) was used to measures the neurite length.

Co-culture assays were performed as described previously (26) with slight modifications. Co-culture studies were performed by adding Neuro-2A cells with density 1000 or 10000 cells per well onto monolayers of DIV7 cerebellar astrocytes enriched culture (70-80% confluence) in 24- or 6-well plate. Co-culture was performed for three days unless otherwise specified. T_3_ (10 nM), T_4_ (10 nM), or rT_3_ (10 nM) were added to the culture medium. Cells were fixed with 4% PFA, followed by blocking with 5% FBS in PBS for 30 minutes. Neuro-2A cells were then immunostained with neuronal marker mouse anti-β-tubulin III antibody (1:200) continued with secondary antibody donkey anti-mouse Alexa Fluor^®^ 405 (1:200). F-actin was stained with CytoPainter Phalloidin-iFluor 594 reagent (Abcam). DAPI was used to stained the cell nuclei. Neuro-2A cells were observed under a laser confocal scanning microscope (Zeiss LSM 880, Carl Zeiss Microscopy GmbH). ImageJ Fiji (NIH) was used to measures the neurite length. Data are showed as the mean ± standard error of the mean (SEM). More than three independent experiments were performed. Results were consistent between each experiment. A representative result from one experiment is shown.

### Immunocytochemistry analysis and F-actin formation

Cultured cells were exposed to 10 nM of T_3_, T_4_, or rT_3_ (Sigma) for one to three days. Cells were washed with PBS three times, then fixed with 4% PFA, and continued with blocking with 5% FBS in PBS. Neuro-2A cells were immunostained with neuronal and presynaptic antibodies, mouse monoclonal anti-β-tubulin III (1:200) (Sigma) and rabbit anti-syn-1 (1:200) (Cell Signaling, MA, USA), continued with secondary antibody donkey anti-mouse Alexa Fluor^®^ 405 (1:200), donkey anti–rabbit Alexa Fluor^®^ 488 conjugate (1:200) (Thermo Fisher Scientific, Inc.), and CytoPainter Phalloidin–iFluor 594 reagent (Abcam). Neuro-2A cells were observed under a laser confocal scanning microscope (Zeiss LSM 880, Carl Zeiss Microscopy GmbH). Filopodia were counted manually. Data are showed as the mean ± standard error of the mean (SEM). More than three independent experiments were performed. Results were consistent between each experiment. A representative result from one experiment is shown.

### RNA interference assay

Primary cultured cerebellar cells or Neuro-2A cells were transfected with siRNAs using lipofectamine RNAiMAX reagent (Thermo Fisher Scientific) according to the manufacturer’s protocol. The siRNA sequences against TRα, TRβ, integrin αv, integrin β3, or negative control DsiRNA (Integrated DNA Technologies, Inc., Coralville, IA, USA) used in this study are listed in **Supplementary Figure 1**. Briefly, 1 nM of DsiRNA, TRα, TRβ, integrin αv, or integrin β3 siRNA in siRNA lipid complexes were incubated for 20 min in the room temperature. Primary cultured cerebellar cells or Neuro-2A cells were culture in 24-, 96-well, or 8 mm diameter wells of chamber slides poly-l-lysine coated dishes until 80% confluences. Then, siRNA lipid complexes were added by dropwise to the well. The quantitative real-time PCR (qRT-PCR) was used to verified the efficacy of siRNA knockdown (**Supplementary Figure 2**). The *SuperPrep* cell lysis and RT kits for qPCR reagent (TOYOBO Bio-Technology, Japan) was used to extract RNA, continued with qRT-PCR using THUNDERBIRD SYBR qPCR Mix (TOYOBO) according to manufacturer’s instructions and a StepOne RT-PCR System (Thermo Fisher Scientific). The primers are listed in **Supplementary Figure 1**. qRT-PCR was performed as follows: denaturation at 95°C for 20 s; amplification at 95°C for 3 s; and 60°C for 30 s (40 cycles). Data are showed as the mean ± standard error of the mean (SEM). More than three independent experiments were performed, using independent RNA preparations to ensure the consistency of results. Glyceraldehyde-3-phosphate dehydrogenase (GAPDH) was used to normalized mRNA levels. Results were consistent between each experiment. A representative result from one experiment is shown.

### Western blot analysis

Cultured cells were homogenized in RIPA buffer (Cell Signaling) containing protease inhibitors (Complete; Roche, IN, USA). Protein concentrations were measured using the Bradford protein assay (Bio-Rad), according to the manufacturer’s instructions. Protein samples (5 µg) was boiling for 5 min, then subjected to 13- or 17-wells of 5%–20% SDS-polyacrylamide Supersep Ace (Wako) gel electrophoresis. Gels that contains separated products were transferred to nitrocellulose membranes, followed by blocking with 5% nonfat dry milk in Tris-buffered saline containing 0.1% Tween 20. Nitrocellulose membranes then incubate overnight with primary antibodies for p-FAK (Y397) (1:1000, Cell Signaling), FAK (1:1000, Cell Signaling), p-ERK1/2 (T202/Y204) (1:1000, Cell Signaling), ERK1/2 (1:1000, Cell Signaling), p-Akt (S473) (1:1000, Cell Signaling), Akt (1:1000, Cell Signaling), and GAPDH (1:1000, Proteintech, IL, USA). After washing with Tris-buffered saline containing 0.1% Tween 20, membranes were incubated with secondary antibody for anti-rabbit or anti-mouse IgG horseradish peroxidase-conjugated (1:3000, Cell Signaling) for one hour at room temperature. Bands of target proteins were detected using an ECL detection system (Wako). ImageJ Fiji (NIH) was used to analyses the bands. Data are showed as the mean ± standard error of the mean (SEM). GAPDH was used as the loading control.

### Statistical analyses

Data were analyzed using GraphPad Prism version 9.3.1 (GraphPad Software, San Diego, USA, www.graphpad.com) or IBM SPSS Statistics 27 for Windows, and showed as the mean ± SEM. Student’s unpaired *t*-test (for two-group comparisons), one-way or two-way analysis of variance (ANOVA) followed by *post hoc* Bonferroni or Turkey multiple comparison tests were performed for data analyses. All *p*-values < 0.05 were considered statistically significant.

## Results

### TH-augmented dendrite arborization of cerebellar Purkinje cells

**Figure 1:**
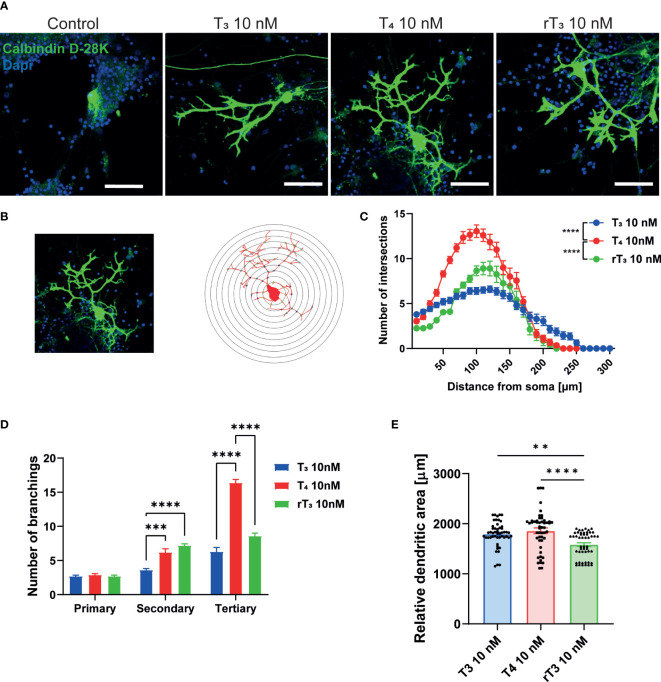
Effect of TH on Purkinje cell dendrite arborization in primary cerebellar culture. Cerebellar cells were cultured for 17 days, followed by an immunocytochemistry analysis of Purkinje cells with Calbindin D-28K (green) and DAPI (blue). **(A)** Representative photomicrographs showing the effects of TH on the morphology of Purkinje cells. **(B)** Schematic diagram of Sholl analysis. **(C)** Quantitative analysis of the effect of TH on the number of dendrite intersections at each concentric circle by Sholl analysis. **(D)** Quantitative analysis of the effect of TH on the number of secondary and tertiary dendrites of Cerebellar Purkinje cells. **(E)** Changes in the dendritic areas of Purkinje cells following TH treatment. ImageJ Fiji software (NIH) was used to quantify the dendritic area of Purkinje cells and Sholl analysis. Bars indicate 50 μm. Data are shown as the mean ± SEM and representative from at least three independent experiments. ****p < 0.0001 and **p < 0.001 indicate statistical significance according to two-way or one-way ANOVA continued with *post hoc* Turkey test. See **Supplementary Table 1**.

TH has been known to regulate cerebellar development for decades. Although the molecular mechanisms underlying the effects of TH on cerebellar development are increasingly well understood, many issues remain to be addressed. Elucidating the actions of TH during dendritic growth and detailed patterning of Purkinje cell arborization will increased knowledge of the intracellular signaling pathways that mediate this process. To examine the effect of TH during dendritogenesis, we performed primary cerebellar cultures. Aside from Purkinje cells, these cultures contained granule cells, astrocytes, and interneurons. Culture cells were maintained in serum-free medium, and dendrite arborization of Purkinje cells was induced by adding T_3_, T_4_, or rT_3_ to the culture medium. After 17 days of culture, cells were fixed and immunostained with an anti-calbindin D–28K antibody to visualize Purkinje cells. T_3_, T_4_, and rT_3_ augmented dendrite arborization of Purkinje cells in a dose-dependent manner (**Supplementary Figure 3**). To quantify dendrite morphology, we performed Sholl analysis (**Figure 1B**). Exposure to T_4_ resulted in a higher number of intersections with dendrites and concentric circles than with exposure to T_3_ or rT_3_, with T_3_ group found to have the most dendrite extensions.

Next, we counted the number of primary, secondary, and tertiary dendrites to evaluate the effects of TH on dendrite branching (**Figure 1D**). The T_4_ group had the highest number of tertiary dendrites compared to the T_3_ and rT_3_ groups. We also measured the dendritic area of Purkinje cells using NIH ImageJ Fiji software, demonstrating significantly higher dendritic areas in the T_3_ and T_4_ group compared to the rT_3_ group. No significant difference was observed between the T_3_ and T_4_ groups (**Figure 1E**). The T_4_ group had higher number of dendritic branches; however, dendritic length was much shorter than the T_3_ group. Therefore, there was no significant difference in dendritic area between groups. This result indicates that TH affects the dendrite arborization of Purkinje cells through several mechanisms of action.

### TR-dependent and TR-independent pathways are required for TH-induced dendrite arborization of Purkinje cells

**Figure 2:**
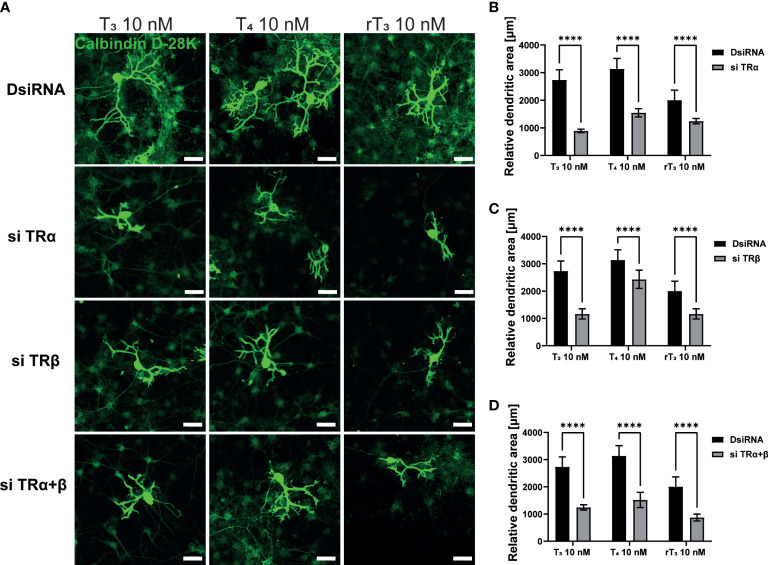
TR deletion impaired TH-augmented dendrite arborization of Purkinje cells. Cerebellar cells were cultured for 17 days, followed by an immunocytochemistry analysis with Calbindin D-28K (green). **(A)** Representative photomicrographs showing the effects of TH on the morphology of Purkinje cells after TR knockdown. Quantitative analysis of the effect of TH after exposure to TRα siRNA **(B)**, TRβ siRNA **(C)**, and siRNA against TRα and TRβ **(D)** on dendritic areas of Purkinje cells following TH treatment. ImageJ Fiji software (NIH) was used to quantify the dendritic area of Purkinje cells. Bars indicate 50 μm. Data are shown as the mean ± SEM and representative from at least three independent experiments. ****p < 0.0001 indicates statistical significance according to two-way or one-way ANOVA continued with *post hoc* Turkey test. See **Supplementary Table 1**.

TH and TR play critical roles during cerebellar development. The majority of TH actions are mediated through nuclear TRs, which can either upregulate or downregulate target gene transcription. Therefore, to examine the involvement of TR in dendritic outgrowth of Purkinje cells, we used siRNA against TRα or TRβ to knock down RNA expression in a primary culture of cerebellar cells. Significant suppression of mRNA levels after siRNA treatment was confirmed (**Supplementary Figure 2**). We also performed double knockdown of both TR mRNAs. Representative photomicrographs of cerebellar Purkinje cells after TH exposure with knockdown of TR are shown (**Figure 2A**). DsiRNA did not significantly affect dendrite arborization of Purkinje cells. However, knockdown of TRα and/or TRβ significantly reduced dendrite arborization of Purkinje cells (**Figure 2B–D**). These results indicate that TRs are essential for dendrite arborization of Purkinje cells. However, it should be noted that we were unable to confirm whether knockdown of TR directly affected Purkinje cell or whether this effect was mediated by other cellular subsets.

**Figure 3:**
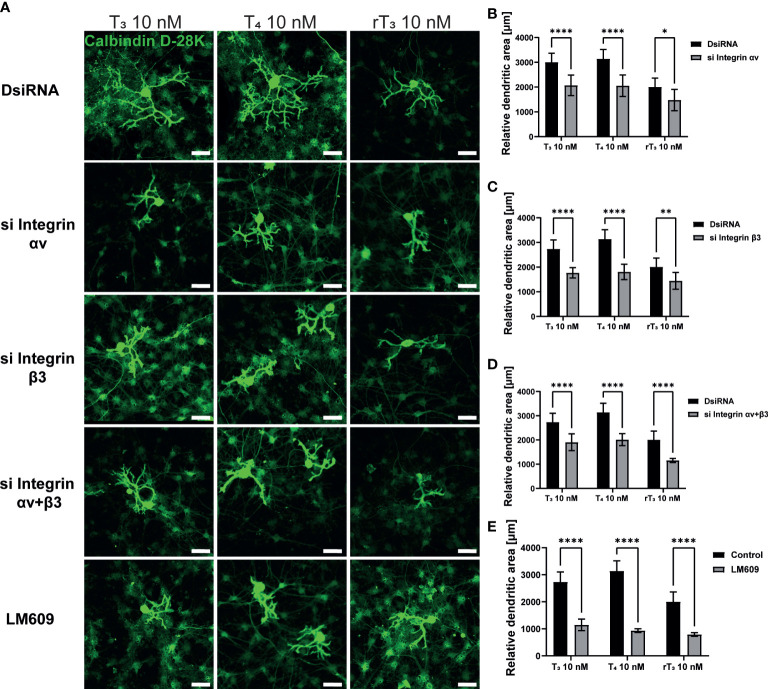
Integrin αvβ3 affected the TH-augmented dendrite arborization of Purkinje cells. Cerebellar cells were cultured for 17 days, followed by an immunocytochemistry analysis with Calbindin D-28K (green). **(A)** Representative photomicrographs showing the effects of TH on the morphology of Purkinje cells after integrin αv or/and β3 knockdown or LM609 treatment. Quantitative analysis of the effect of TH after exposure to integrin av siRNA **(B)**, integrin b3 siRNA **(C)**, integrin av and b3 siRNA **(D)**, and LM609 2 μg/mL **(E)** on dendritic areas of Purkinje cells following TH treatment. ImageJ Fiji software (NIH) was used to quantify the dendritic area of Purkinje cells. Bars indicate 50 μm. Data are shown as the mean ± SEM and representative from at least three independent experiments. ****p < 0.0001, **p < 0.01 and *p < 0.05, indicate statistical significance according to two-way or one-way ANOVA continued with *post hoc* Turkey test. See **Supplementary Table 1**.

In addition to TR, the integrin αvβ3 may serve as a membrane receptor of TH. We examined the effects of integrin αvβ3 on dendritic outgrowth of Purkinje cells in primary cerebellar culture by siRNA transfection and co-exposure with integrin αvβ3 monoclonal antibody (mAb), LM609. LM609 is a monoclonal antibody against integrin αvβ3, and function as an allosteric inhibitor of integrin αvβ3, by reducing complex formation of integrin αvβ3and TH (27). Significant suppression of mRNA expression levels after siRNA treatment was confirmed (**Supplementary Figure 2**). Representative photomicrographs of Purkinje cells after TH exposure with knockdown of integrin αvβ3 or co-exposure with LM609 are shown (**Figure 3A**). Knockdown of integrin αv or β3 subunits or double knockdown of integrin αvβ3 significantly reduced dendrite arborization of Purkinje cells (**Figure 3B–D**). Furthermore, co-exposure with the integrin αvβ3 mAb, LM609 (2 µg/mL), also reduced TH-induced dendrite arborization of Purkinje cells. These results indicate that the TR-independent signaling pathway *via* integrin αvβ3 also plays a role in cerebellar development, at least in part.

### TH-induced neurite outgrowth in Neuro2A cells *via* TRα and integrin αvb3

**Figure 4:**
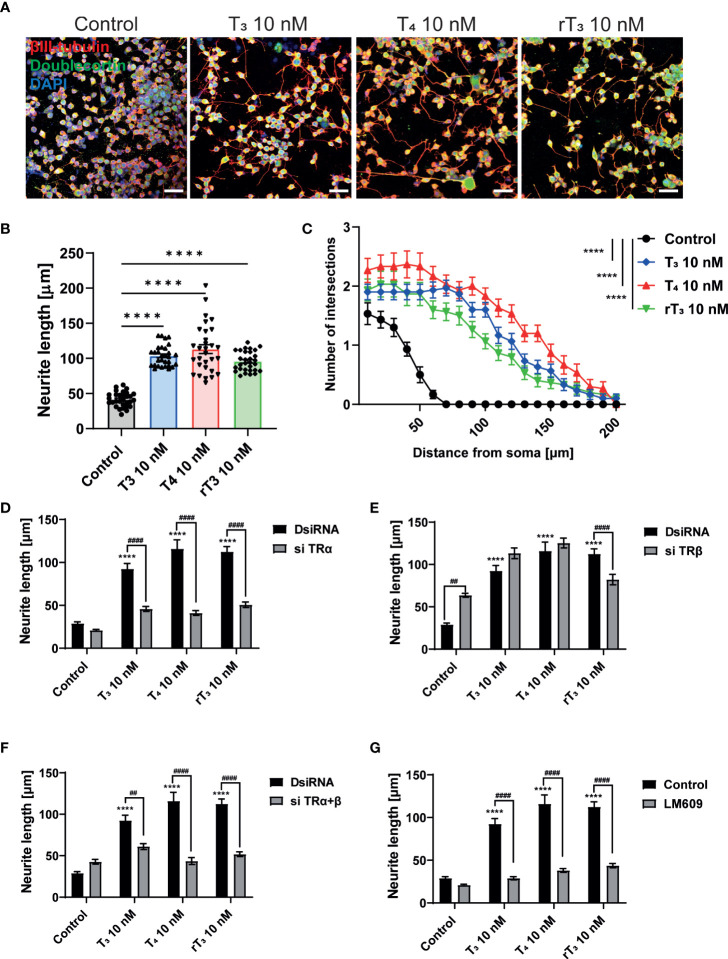
TRα and integrin αvβ3 affect neurite outgrowth induced by TH in neuronal-derived Neuro-2A cells. Cells were induced to differentiate by serum starvation for one to three days, followed by immunofluorescence analysis with β-tubulin III (red), doublecortin (green), and DAPI (blue). **(A)** Representative photomicrographs showing the effects of TH on the differentiation of Neuro-2A cells. **(B)** Changes in neurite lengths after TH exposure. **(C)** Quantitative analysis of the effect of TH on the number of neurite intersections at each concentric circle by Sholl analysis. Neurite length and Sholl analysis were quantified using ImageJ Fiji software (NIH). Bars indicate 50 μm. Quantitative analysis of the effect of TH after exposure to TRa siRNA **(D)**, TRb siRNA **(E)**, TRa and TRb siRNA **(F)**, and LM609 2 μg/mL **(G)** on neurite length in Neuro-2A cells. ****p < 0.0001, indicates statistical significance according to two-way or one-way ANOVA continued with *post hoc* Turkey test compared to control. ^####^ *p* < 0.0001 and ^##^ *p* < 0.01, indicate statistical significance according to two-way ANOVA continued with *post hoc* Bonferroni’s test. See **Supplementary Table 1**.

Primary cerebellar cultures contain several different subsets of cells. Thus, it is difficult to clarify whether the TH action is mediated by direct effects on the Purkinje cells or by cell-cell interactions. In order to examine the mechanism of action of TH during development, we used a Neuro-2A cell differentiation model. Neuro-2A differentiation was induced by reducing the concentration of FBS (TH–depleted) in culture medium to 1%. Cells were then immunostained with neuronal marker, anti-β-tubulin III for mature neuron and anti-doublecortin (DCX) for immature neuron or neurogenesis marker (25, 28). TH (T_3_, T_4_, and rT_3_) at a concentration of 10 nM enhanced neurite outgrowth (**Figure 4A, B**). Neurite quantification by Sholl analysis demonstrated TH exposure increased neurite outgrowth (**Figure 4C**). Using this cell type, we further examined the involvement of TR and integrin αvβ3 during neuritogenesis using siRNA and the integrin αvβ3 mAb LM609. Significant suppression of mRNA expression levels after siRNA treatment was confirmed (**Supplementary Figure 2**). We found that knockdown of TRα or double knockdown of TR significantly reduced TH-induced neurite outgrowth in Neuro-2A cells, whereas no significant difference was observed after knockdown of TRβ, except in the rT_3_ group. Surprisingly, knockdown of TRβ enhanced neurite outgrowth in the control group (**Figure 4D–F**). Co-exposure to TH with LM609 (**Figure 4G**) or knockdown of integrin αvβ3 (**Supplementary Figure 4**) also reduced TH-induced neuritogenesis. These results demonstrate that integrin αvβ3, in addition to TRα, is involved in the neuritogenesis of Neuro-2A cells.

### *Neuron*-*glia interaction enhanced TH*-*induced neuritogenesis*

**Figure 5:**
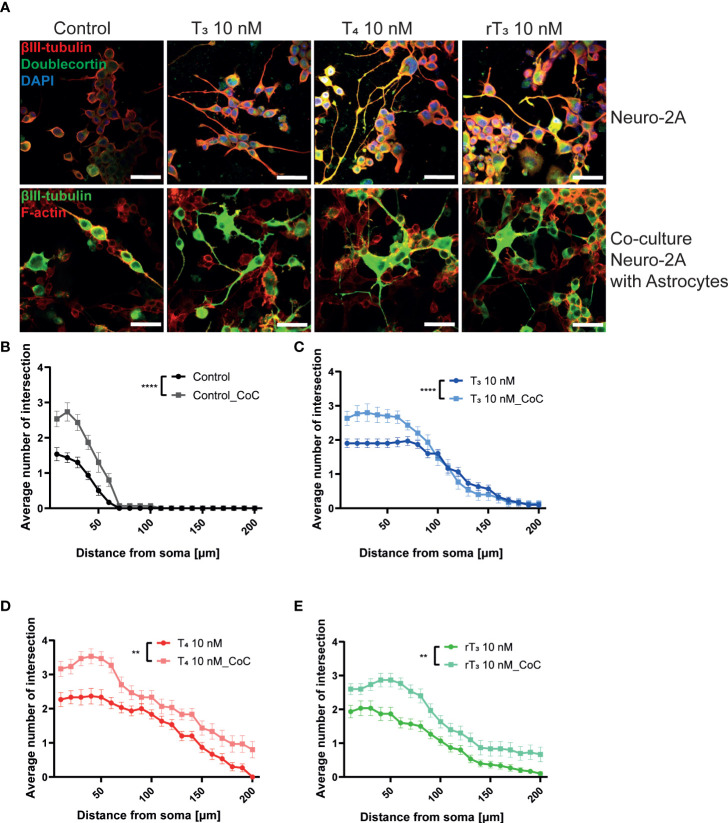
Co-culture with astrocytes enhanced TH-augmented neurite length in Neuro-2A cells. **(A)** Representative photomicrographs showing the effects of TH, with or without co-culture with astrocytes, on the differentiation of Neuro-2A cells. Neuro-2A cell differentiation was induced by serum starvation for one to three days, followed by immunostaining with β-tubulin III (red), doublecortin (green), and DAPI (blue) (upper panel) or β-tubulin III (green) and F-actin (red) (lower panel). Quantitative analysis of the effect of co-culture with astrocytes **(B)** and exposure to T_3_ **(C)**, T_4_ **(D)**, and rT_3_ **(E)** on the number of intersections of neurite at each concentric circle by Sholl analysis. ****p < 0.0001 and **p < 0.01 indicate statistical significance according to two-way ANOVA. See **Supplementary Table 1**.

**Figure 6:**
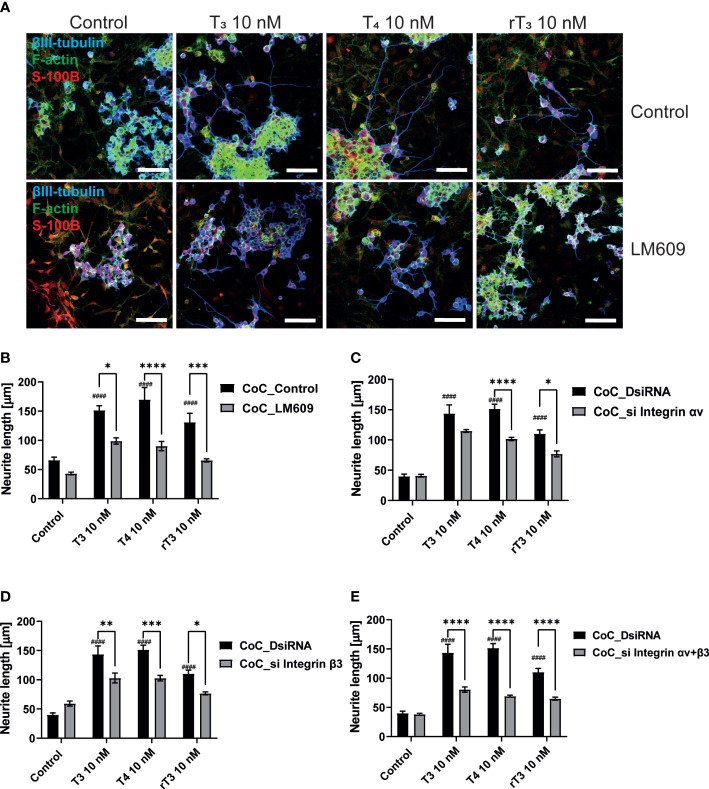
Integrin αvβ3 affect neurite outgrowth induced by TH in co-culture of astrocytes and Neuro-2A cells. **(A)** Representative photomicrographs showing the effects of TH and co-exposure with LM609 in the co-culture of astrocytes and Neuro-2A cells, on the differentiation of Neuro-2A cells. Neuro-2A cell differentiation was induced by serum starvation for one to three days, followed by immunostaining with β-tubulin III (blue), F-actin (green), and S100B (astrocytes marker) (red). Quantitative analysis of the effect of TH after exposure to LM609 **(B)**, integrin av siRNA **(C)**, integrin b3 siRNA **(D)**, and integrin av and b3 siRNA **(E)**, on neurite length in Neuro-2A cells. Neurite length was quantified using ImageJ Fiji software (NIH). Bars indicate 50 μm. ****p < 0.0001, ***p < 0.001 and **p < 0.01 *p < 0.05, indicates statistical significance according to two-way ANOVA continued with *post hoc* Turkey test compared to control. ^####^ *p* < 0.0001, indicate statistical significance according to two-way ANOVA continued with *post hoc* Turkey test. See **Supplementary Table 1**.

The interaction between neurons, astrocytes, and microglia has an essential role during development, mediating metabolic processes, supplying nutrients, and improving brain metabolism and function. During development, the surrounding environment and cell-to-cell communication between neurons, astrocytes, microglia, endothelial cells, and oligodendrocytes greatly influences cellular maturation (29–35). Due to this close relationship, slight environmental variations can cause changes in the phenotype of cells, gene expression, and intracellular dynamics of Ca^2+^ in the brain (35). We performed a co-culture study to examine the effects of TH exposure on neuron-glia interactions. We cultured Neuro-2A cells in a cerebellar astrocytes-enriched culture for 3–5 days. Neuro-2A cells were then immunostained with anti-β-tubulin III and anti-doublecortin (DCX) to examine the neurite outgrowth. In the co-culture of Neuro-2A with astrocytes, doublecortin was stained both Neuro-2A cells and soma of the astrocytes. Therefore, immunostainings with anti-β-tubulin III and F-actin were used instead of doublecortin in the co-culture study. The F-actin was used to examine the cell-cell interaction of Neuro-2A cells and astrocytes. Representative photomicrographs of Neuro-2A cells and co-culture Neuro-2A with astrocytes are shown (**Figure 5A**). We quantified the branching and elongation of neurites using Sholl analysis. Co–culture of Neuro-2A with astrocytes enhanced TH-induced neurite outgrowth, including branching and elongation (**Figure 5B–D**). In addition, co-exposure with LM609 and knockdown of integrin αvβ3 in co-culture study significantly reduced TH-induced neuritogenesis (**Figure 6**). These results indicate that integrin αvβ3 is necessary in the neuron-glia interaction to induces neuritogenesis.

### Augmentation of neurite outgrowth was associated with F-actin formation and activation of FAK/Akt/ERK signaling pathways

**Figure 7:**
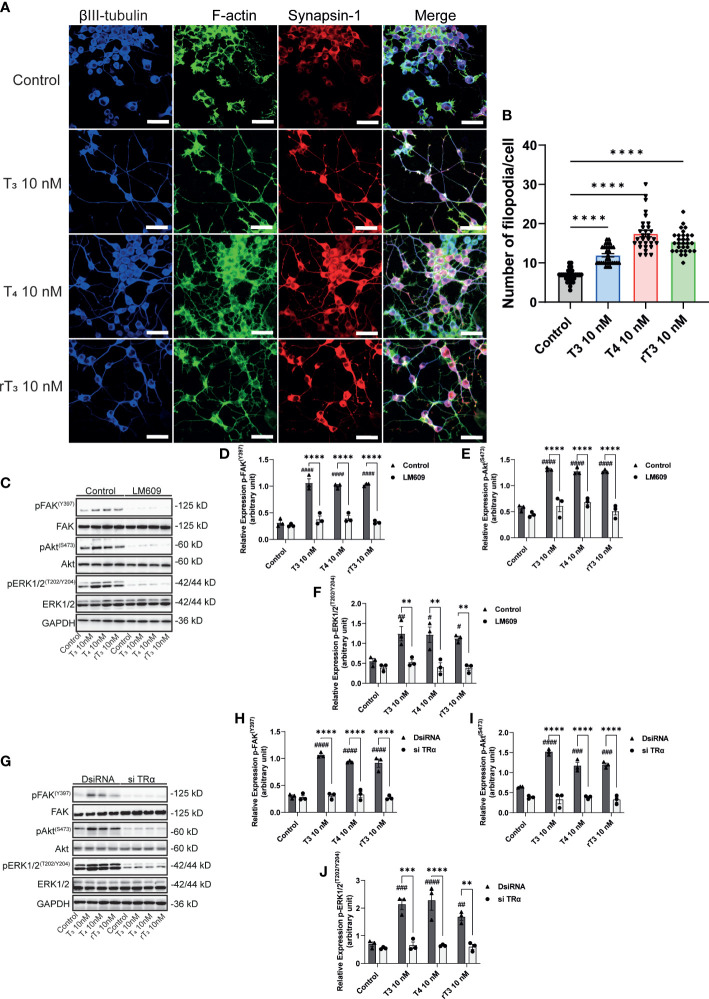
Effect of TH on F-actin formation and phosphorylation of FAK, Akt, and ERK1/2. A. Representative photomicrographs showing the effects of TH on the filopodia formation in Neuro-2A cells. Differentiation of Neuro-2A cells was induced by serum starvation for one to three days, followed by immunostaining with β-tubulin III (blue), F-actin (green), and synapsin-1 (red). **(B)** Quantitative analysis of filopodia formation in Neuro-2A cells after TH treatment. **(C)** Representative images of blots for pFAK, FAK, pAkt, Akt, pERK1/2, ERK1/2, and GAPDH levels following TH treatment and co-exposure to 2 µg/mL LM609. Quantitative analysis of the effect of TH on protein expression levels of FAK (Y397) **(D)**, Akt (S473) **(E)**, and ERK1/2 (T202/Y204) **(F)**. **(G)** Representative images of blots for pFAK, FAK, pAkt, Akt, pERK1/2, ERK1/2, and GAPDH levels in response to TH treatment after deletion of TRα. Quantitative analysis of the effect of TH on FAK (Y397) **(H)**, Akt (S473) **(I)**, and ERK1/2 (T202/Y204) protein expression levels **(J)**. ImageJ Fiji software (NIH) was used to quantify the blots. Data are shown as the mean ± SEM and representative from at least three independent experiments. ^####^ *p* < 0.0001, ^###^ *p* < 0.001, ^##^ *p* < 0.01 and ^#^ *p* < 0.05 indicate statistical significance according to two-way ANOVA continued with *post hoc* Turkey test compared to control. ****p < 0.0001, ***p < 0.001 and **p < 0.01 indicate statistical significance according to two-way or one-way ANOVA continued with *post hoc* Turkey test. See **Supplementary Table 1**.

To further elucidate the molecular mechanisms underlying TH-induced neuritogenesis, we performed immunostaining of synapsin-1 and F-actin to observe F-actin formation. TH reportedly influences *in vitro* actin polymerization through an unknown mechanism. The interaction of integrin αvβ3 with its ligands induces cellular polarization and formation of filopodia at the leading edge. We found that TH exposure enhanced the co-localization of synapsin-1 protein with F-actin (**Figure 7A**). TH exposure also increased the number of filopodia in Neuro-2A cells. In addition, we also performed Western blot analysis after co-exposure with the integrin αvβ3 mAb, LM609, or knockdown of TRα. We found that TH exposure increased phosphorylation levels of FAK (Y397), Akt (S473), and ERK1/2 (T202/Y204). Co-exposure with LM609 or knockdown of TRα significantly reduced the phosphorylation levels (**Figure 7C–J**). These results indicate that integrin αvβ3 and TRα are involved in the phosphorylation and activation of FAK, Akt, and ERK1/2, leading to F-actin remodeling during neuritogenesis.

## Discussion

Herein, we examined the effects of TH (T_3_, T_4_, and rT_3_) on dendrite arborization of Purkinje cells and neurite outgrowth of neuronal-derived clonal cells, Neuro-2A. The results of the present study demonstrate that TH derivatives have differing effect on dendritogenesis in Purkinje cells, particularly in dendritic branching and the detailed patterning of Purkinje cells. TH derivatives induced dendrite arborization in Purkinje cells at a range of doses. Furthermore, both TH-TR and TH-integrin αvβ3 interactions are required for dendrite arborization of Purkinje cells. TH also induced neurite outgrowth in Neuro-2A cells through TRα- and integrin αvβ3-mediated signaling pathways. In addition, co-culture of Neuro-2A with a cerebellar astrocyte-enriched culture also increased neurite outgrowth in Neuro-2A cells. TH-TR and TH-integrin αvβ3 interactions also increased the phosphorylation levels of FAK, Akt, and ERK1/2 in Neuro-2A cells. Knockdown of TRα or co-exposure with LM609 significantly attenuated this effect. TH exposure also increases filopodia formation, which is colocalized with presynaptic protein synapsin1. Our results have demonstrated TH’s novel mechanism of action in promoting dendritogenesis and neuritogenesis *via* TRα and integrin αvβ3 signaling pathway by modulating phosphorylation of downstream signal proteins and F-actin remodeling.

The cerebellum consists of various cell types, including Purkinje cells, granule cells, basket cells, stellate cells, and astrocytes, and is comprised of a cytoarchitecture array of stripes and zones (8). The interaction between neurons, astrocytes, and microglia have essential roles for normal cerebellar development, by supplying nutrients, and improving metabolism and function, thereby promoting cellular maturation (29–35). Due to such close relationships, slight environmental variations can cause changes in the phenotype of cells, gene expression, and intracellular dynamics of Ca^2+^ in the brain (35). Early exposure of dominant negative TRα1^L400R^ in specific cell type-autonomously affects dendrite arborization of Purkinje cells (36). TH exposure in astrocytes induces EGF release that affects cerebellar neuritogenesis and cell proliferation (37). TH also increases the growth of astrocytes and granule cells, leading to enhanced dendrite arborization of Purkinje cells (38). Herein, we found that TH exposure induces neurite outgrowth in a co-culture of neurons and astrocytes (**Figure 5**). Furthermore, as primary cultures of cerebellar cells contain various cellular subsets, cell-cell interactions may enhance the effects of TH. Hence, indirect effects of TH from other subset of cells to Purkinje cells may also alter the dendrite arborization. Thus, determining the effects of TH on specific cell types both *in vitro* and *in vivo* remains a major goal for studies investigating the role of TH during cerebellar development.

TH is known to play an essential role during cerebellar development, including Purkinje cells dendritogenesis. The absence of TH during the first postnatal weeks in rodents causes hypoplasia and aberrant dendrite arborization of Purkinje cells. Consequently, congenital hypothyroidism results in neurological symptoms such as ataxia and poor fine motor movement. However, whether TH acts directly on Purkinje cells or indirectly through other cellular subset such as granule cells or astrocytes interaction remains poorly understood. A previous study reported that both T_3_ and T_4_ promoted the dendritic arborization of Purkinje cells (38). Furthermore, T_4_-treated cultures also affect the growth of astrocytes and granule cells, which may be necessary for normal dendritic arborization of Purkinje cells (38). Our study is the first to report the effect of rT_3_ on dendrite arborization of Purkinje cells. We found that rT_3_ exposure induced dendrite arborization of Purkinje cells with lesser potency than T_4_ or T_3_ (**Figure 1** and **Supplementary Figure 3**). However, T_4_ and rT_3_ induced greater dendritic branching compared to T_3_. These results indicate that T_4_ and rT_3_ may activate different signaling pathways to enhance dendrite arborization in Purkinje cells.

The majority of TH actions are mediated by interactions with TRs that can either upregulate or downregulate target gene transcription (39). Both TRs isoforms are highly expressed in the cerebellum, including Purkinje cells. Purkinje cells in TRα or TRβ knockout/mutant mice have aberrant dendrite arborization (36, 40–42). Although the signal transduction pathway responsible for these effects has yet to be characterized in detail, it appears that both TRα and TRβ play essential roles during Purkinje cell development. In corroboration with these previous studies, we found that knockdown of TRα and/or TRβ significantly reduced TH–mediated dendrite arborization of Purkinje cells (**Figure 2**). However, only TRα affected neuritogenesis in neuronal-derived Neuro-2A cells *via* phosphorylation and activation of protein kinases (**Figure 4**, **6**). These results may be due to the low expression levels of TRβ in Neuro-2A cells, or the effect of TH *via* TRβ may be mediated by granule/glial cell interactions with Purkinje cells. Thus, further studies are required to clarify the exact processes underlying for these differences.

TH can alter the expression of a wide variety of genes, either directly or indirectly, in the developing cerebellum, including neurotrophin-3 (NT-3), brain-derived neurotrophic factor, retinoid-receptor-related orphan receptor-α (RORα), and epidermal growth factor (EGF) (10, 37, 43). T_3_ exposure also promotes dendritic outgrowth of Purkinje cells through induction of peroxisome proliferator-activated receptor-gamma (PPARγ) co-activator 1α (PGC–1α) expression (44). Furthermore, T_3_ treatment also induced dendrite arborization of the Purkinje cell by increasing the mRNA and protein expression levels of RORα (43). Although most previous studies have used T_3_ to examine TH-induced gene expression, T_4_ and rT_3_ may also play important roles. T_4_ enhanced dendrite arborization of Purkinje cells (41, 45). Cerebellar developmental defects such as impaired neurogenesis or delayed migration of granule cells can be rescued by T_4_ or rT_3_ but not T_3_ (13, 46). Exposure to T_4_ or rT_3_ during cerebellar development increase protein transport, protein kinase activation, and restores laminin deposition (13, 46, 47). These results indicate TH derivatives (T_3_, T_4_, and rT_3_) differentially activate various signaling pathways necessary for normal cerebellar development.

In addition to TR, we found that TH-mediated dendritogenesis and neuritogenesis were mediated by integrin αvβ3, a membrane receptor for TH. The integrin αvβ3 has been reported to regulate neurite outgrowth and synaptogenesis in several brain regions (17, 48, 49). A recent study found that integrin β3-mediated changes in dendritic branching of cerebral pyramidal neurons, rather than changes in average segment length (22). These effects may be caused by F-actin and protein kinase modulation, particularly FAK (19, 50). In addition, hypothyroid neonatal rodents have abnormally low cerebellar F-actin levels, which can be restored to normal levels within three hours by a single injection of either T_4_ or rT_3_ (13, 47). Although T_3_ has little effect on F-actin, T_3_ has been shown to activate PI3K and ERK1/2, leading to induced cerebellar neuritogenesis (37). In the present study, we found that TH increased filopodia formation and activated FAK, Akt, and ERK1/2 (**Figure 6**). TH-integrin αvβ3 interactions induced the activation of protein kinases to modulate F-actin remodeling, leading to increased dendritic branching and synaptogenesis. Although further studies are required to fully elucidate the effects of TH signaling through integrin αvβ3 on dendritic complexity and spine density, the results of the present study clearly demonstrate that TH signaling *via* integrin αvβ3 is required to induce dendrite arborization.

Deiodinases mediate the balance of local TH levels by controlling conversion of TH. They catalyse the removal of inner or outer ring iodine atoms in equimolar proportions to generate T_3_, rT_3_, or T_2_, depending on the substrate (51, 52). It has been reported that TH mediates gene expression and enzyme activity, of type 1, 2, and 3 deiodinases (51, 53–55). However, whether these effects are mediated through nuclear TR or the others pathways, it remains unclear. In this study, we found that TH exposure increased mRNA levels of type 1, 2, and 3 deiodinases in primary cerebellar culture. However, only in *Dio1*, LM609 significantly reduced T_4_- and rT_3_-enhanced mRNA levels. There was no significant difference in the mRNA levels of *Dio2* and *Dio3* by LM609 (**Supplementary Figure 5**). Since D2 and D3 play a major role in the TH metabolism in the brain (3, 12), we considered that the alteration of deiodinase may not play a major role in integrin-mediated dendritogenesis. In addition, rT_3_ and T_2_ also have been reported affect TR-independent pathways, especially in the mitochondria (16, 56). Nevertheless, limited studies regarding rT_3_ and T_2_ effects on the brain are available. These issues may become a new perspective regarding TH action in the neuritogenesis and dendritogenesis. Hence, further study is necessary to clarify the signaling pathways of each TH derivatives, as well as those to control deiodinase activity.

In conclusion, we demonstrated TR and integrin αvβ3-mediated signaling pathways play essential roles in TH-mediated dendritogenesis. The effects of TH are at least in part mediated by activation of PI3K and ERK1/2 pathways, leading to F-actin polymerization. Although further studies may be required, to elucidate the exact underlying mechanisms, the results of the present study contribute greatly to understanding of the mechanisms of TH action during brain development particularly in the cerebellum.

## Data availability statement

The raw data supporting the conclusions of this article will be made available by the authors, without undue reservation.

## Ethics statement

The animal study was reviewed and approved by Animal Care and Experimentation Committee, Gunma University.

## Author contributions

WA designed and performed the experiments, analyzed the results, and wrote the manuscript. WM, and IA, supervised the project. NK designed the experiments, evaluated the data, and revised the manuscript. NK supervised the whole project related to thyroid hormone action and brain development performed in the Department of Integrative Physiology, Gunma University Graduate School of Medicine. All authors contributed to the article and approved the submitted version.

## Funding

This work was supported by Grants-in-Aid for Scientific Research (nos. 18H03379 to NK, 16K00557 to WM, and 18J23449 to WA) from the Japanese Ministry of Education, Culture, Sports, Science and Technology (MEXT).

## Acknowledgments

We would like to thank the Department of Integrative Physiology staff, Graduate School of Medicine, Gunma University, Japan.

## Conflict of interest

The authors declare that the research was conducted in the absence of any commercial or financial relationships that could be construed as a potential conflict of interest.

## Publisher’s note

All claims expressed in this article are solely those of the authors and do not necessarily represent those of their affiliated organizations, or those of the publisher, the editors and the reviewers. Any product that may be evaluated in this article, or claim that may be made by its manufacturer, is not guaranteed or endorsed by the publisher.
