# Supplementary material for: Involvement of integrin αvβ3 in thyroid hormone-induced dendritogenesis
Source: Front Endocrinol (Lausanne). 2022 Aug 22;13:938596. doi: 10.3389/fendo.2022.938596 (PMC9441609; doi:10.3389/fendo.2022.938596)
Supplement: Supplementary file 1 [file DataSheet_1.pdf]

Supplementary Information

Involvement of integrin αvβ3 in thyroid hormone-induced dendritogenesis

Winda Ariyani[1,2*], Wataru Miyazaki[2,3], Izuki Amano[2], Noriyuki Koibuchi[2*].

[1] International Research Fellow of Japan Society for the Promotion of Science, 5-3-1 Kojimachi, Chiyoda-ku, Tokyo 102-0083, Japan, winda@gunma-u.ac.jp.

[2] Department of Integrative Physiology, Gunma University Graduate School of Medicine, 3-39-22 Showa-machi Maebashi, Gunma 371-8511, Japan, nkoibuch@gunma-u.ac.jp.

[3] Department of Bioscience and Laboratory Medicine, Hirosaki University Graduate School of Health Science, 66-1 Hon-cho Hirosaki, Aomori 036-8564 Japan, miya@hirosaki-u.ac.jp.

Running title: TH modulates integrin αvβ3-mediated dendritogenesis

*Correspondence authors:

Winda Ariyani, PhD.

Noriyuki Koibuchi, MD., PhD.

Department of Integrative Physiology, Gunma University, Graduate School of Medicine.

3-39-22 Showa-machi Maebashi, Gunma 371-8511 Japan.

Phone No.: +81-27-220-7923

FAX No.: +81-27-220-7926

E-mail address: winda@gunma-u.ac.jp; nkoibuch@gunma-u.ac.jp

Supplementary Figure 1. List of siRNA target sequences (Table 1) and qRT-PCR primer sequences (Table 2).

Supplementary Figure 2. mRNA expression levels of TR or integrin αvβ3 after siRNA treatment. A. Changes in TRα or TRβ mRNA expression levels in primary cerebellar culture after siRNA transfection. B. Changes in integrin αv or integrin β3 mRNA expression levels in primary cerebellar culture after siRNA transfection. C. Changes in TRα or TRβ mRNA expression levels in Neuro-2A cells after siRNA transfection. All experiments were repeated three times, using independent RNA preparations to confirm the consistency of the results. mRNA levels were normalized against GAPDH. Data are shown as the mean ± SEM. ****$p < 0.0001$ indicates statistical significance according to Student's unpaired t-test. See Supplementary Table 1.

Supplementary Figure 3. Effect of TH on Purkinje cell dendrite arborization in primary cerebellar culture. Cerebellar cells were cultured for 17 days, followed by an immunocytochemistry analysis with Calbindin D-28K (green) and DAPI (blue). A. Representative photomicrographs showing the effects of TH on the morphology of Purkinje cells. Changes in the dendritic areas of Purkinje cells following T_3 (B), T_4 (C), or rT_3 (D) treatment. Dendritic areas were quantified using ImageJ Fiji software (NIH). Bars indicate 50 μm. Data are shown as the mean ± SEM. ****$p < 0.0001$ and **$p < 0.001$ indicate statistical significance according to one-way ANOVA continued with post hoc Turkey's test. See Supplementary Table 1.

Supplementary Figure 4. Integrin αvβ3 affect neurite outgrowth induced by TH in neuronal-derived Neuro-2A cells. Cells were induced to differentiate by serum starvation for one to three days, followed by immunofluorescence analysis with β-tubulin III (red), doublecortin (green), and DAPI (blue). Quantitative analysis of the effect of TH after exposure to integrin αv siRNA (A), integrin β3 siRNA (B), integrin αv and β3 siRNA (C), on neurite length in Neuro-2A cells. Neurite length was quantified using ImageJ Fiji software (NIH). Bars indicate 50 μm. Data are shown as the mean ± SEM. ****$p < 0.0001$, indicates statistical significance according to two-way or one-way ANOVA continued with post hoc Turkey's test compared to control. ####$p < 0.0001$ and ##$p < 0.01$, indicate statistical significance according to two-way ANOVA continued with post hoc Turkey's test. See Supplementary Table 1.

Supplementary Figure 5. mRNA levels of *Dio1*, *Dio2*, or *Dio3* after co-exposure with LM609. Changes in *Dio1* (A), *Dio2* (B), or *Dio3*(C), mRNA levels in primary cerebellar culture after co-exposure with LM609. All experiments were repeated three times, using independent RNA preparations to confirm the consistency of the results. mRNA levels were normalized against GAPDH. Data are shown as the mean ± SEM. ****$p < 0.0001$, ***$p < 0.001$ and **$p < 0.01$ *$p < 0.05$, indicates statistical significance according to two-way ANOVA continued with post hoc Turkey's test. See Supplementary Table 1.

Supplementary Table 1. Statistical Analysis Results.
