# Supplementary material for: Involvement of integrin αvβ3 in thyroid hormone-induced dendritogenesis
Source: Front Endocrinol (Lausanne). 2022 Aug 22;13:938596. doi: 10.3389/fendo.2022.938596 (PMC9441609; doi:10.3389/fendo.2022.938596)
Supplement: Supplementary file 2 [file Image_1.pdf]

Supplementary Figure 1.

Table 1. siRNA target sequences.

Gene	Sense	Antisense
DsiRNA Controls	CGUUAAUCGCGUAUAAUACGCGUAT	AUACGCGUAUUAUACGCGAUUAACGAC
Integrin αv (Mouse)	ACAGUUGACUUCAUAAGC	UUCAGUAGCUUAUGAAGU
Integrin β3 (Mouse)	CUAGGCAAGAACAUUACC	AUCAGUUGGUAAUGUUCU
TRα (Mouse)	GCAUCCUGGAUGGAAUUC	UUCACUUCAAUUCCAUCC
TRβ (Mouse)	GGCAUCAGGUCCUAAAAU	UAAGCUUAUUUUAGGACC

Table 2. Primer sequences.

Gene	Sense	Antisense
Itgav (Mouse)	TGTGAAGGCGCAGAATCAAG	AATGCACAGGACAGTCTTGC
Itgb3 (Mouse)	GGCAAAAACGCCGTGAATTG	TTCCACCACATAGAGGACTGC
Thra (Mouse)	CGCTTCAAGAAGTGCATTGC	TCAATCAGCTTGCGTTTGGC
Thrb (Mouse)	ACAAGCACCCATCGTGAATG	TGGCAGCTCACAAAACATGG
Dio1 (Mouse)	GCTGGCAGAGACTGGAAGAC	CCTGCTGCCTTGAATGAAAT
Dio2 (Mouse)	TGTGTCTGGAACAGCTTCCTC	ACGTGCACCACACTGGAAT
Dio3 (Mouse)	TCAGACGACAACCGTCTGTG	AAAATTGAGCACCAACGGGC
Gapdh (Mouse)	TGCGACTTCAACAGCAACTC	ATGTAGGCCATGAGGTCCAC
