# Supplementary figures and images for: Involvement of integrin αvβ3 in thyroid hormone-induced dendritogenesis
Source: Front Endocrinol (Lausanne). 2022 Aug 22;13:938596. doi: 10.3389/fendo.2022.938596 (PMC9441609; doi:10.3389/fendo.2022.938596)

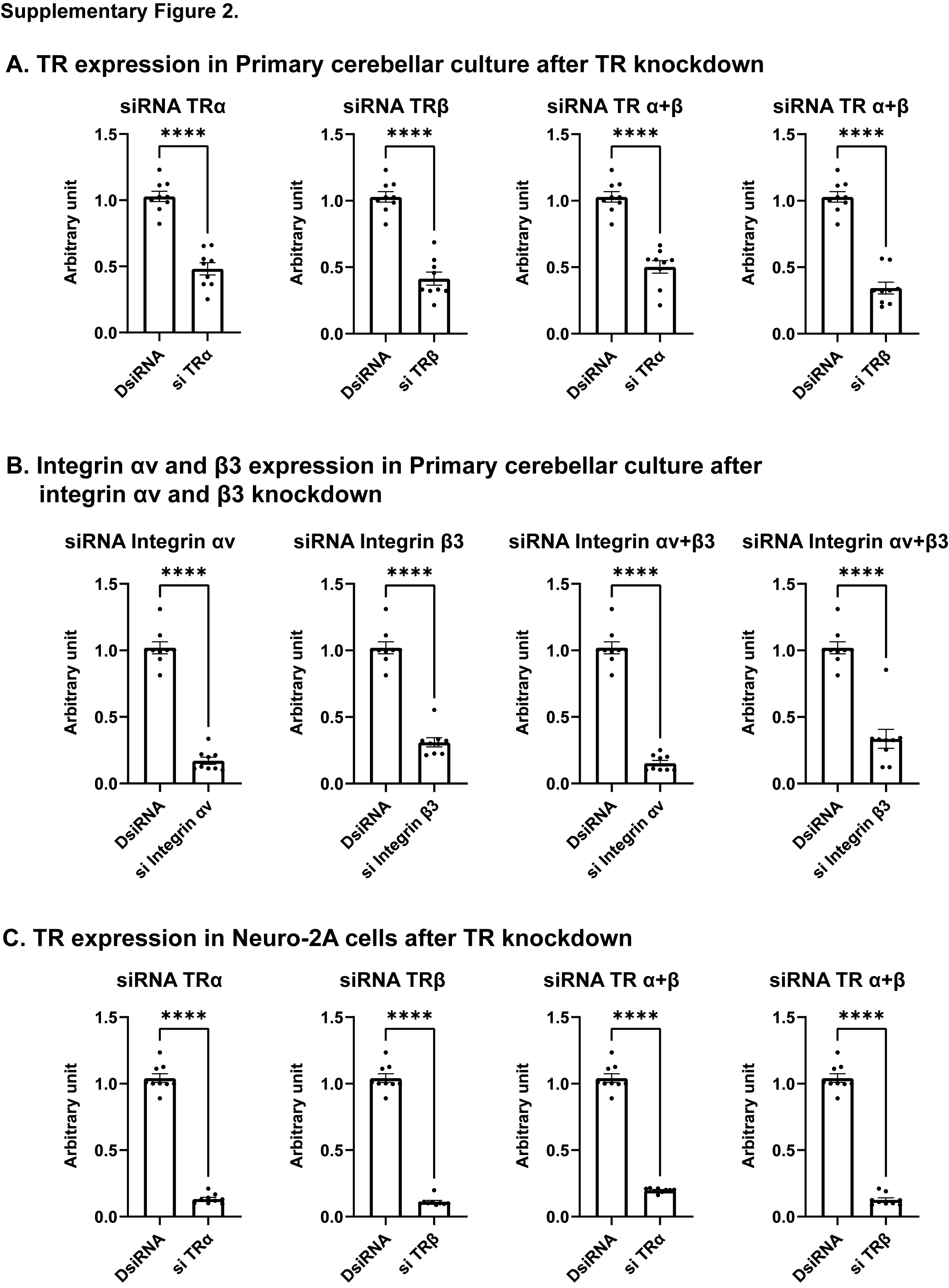

Supplement: Supplementary file 3 [file Image_2.tif]

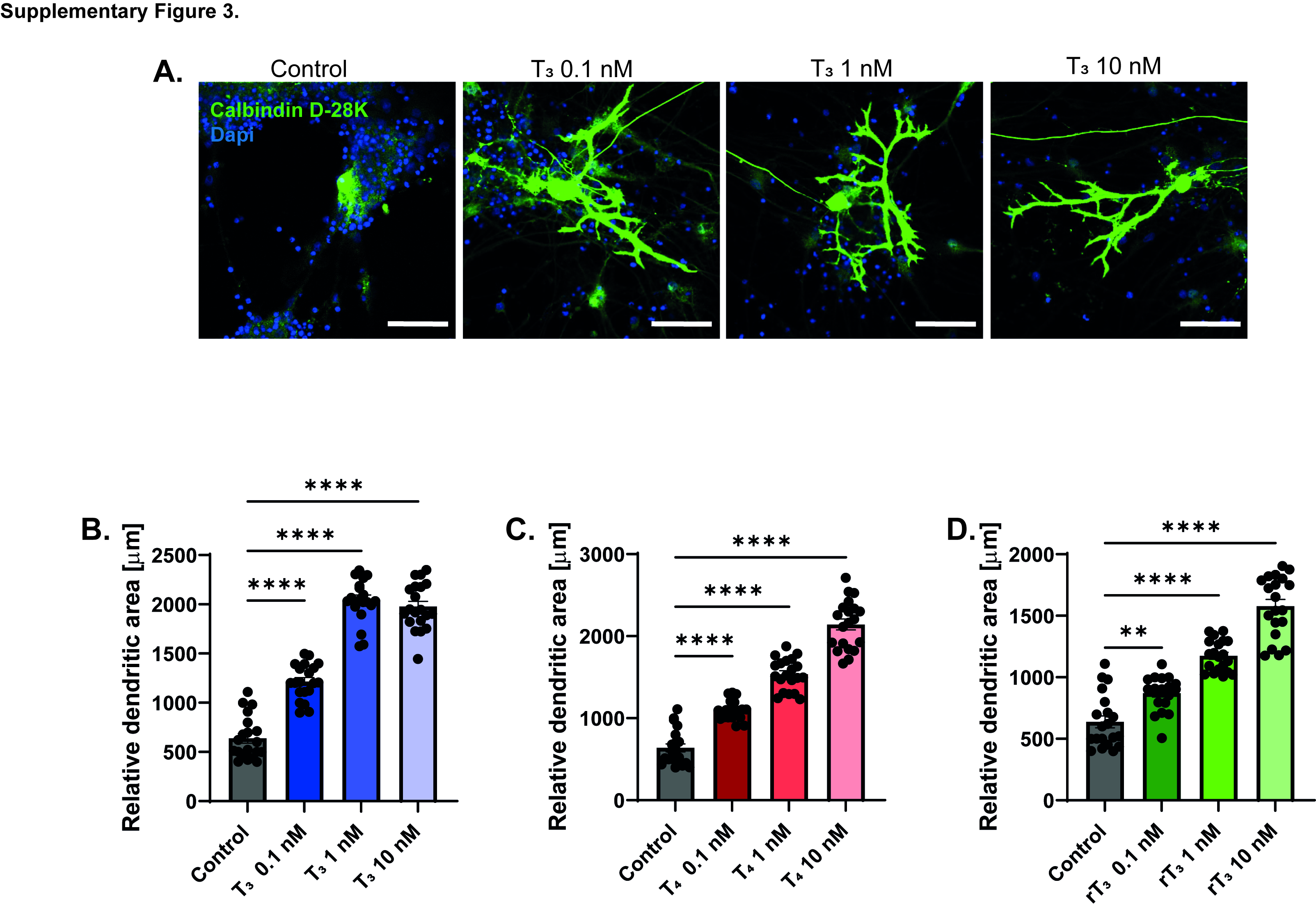

Supplement: Supplementary file 4 [file Image_3.tif]

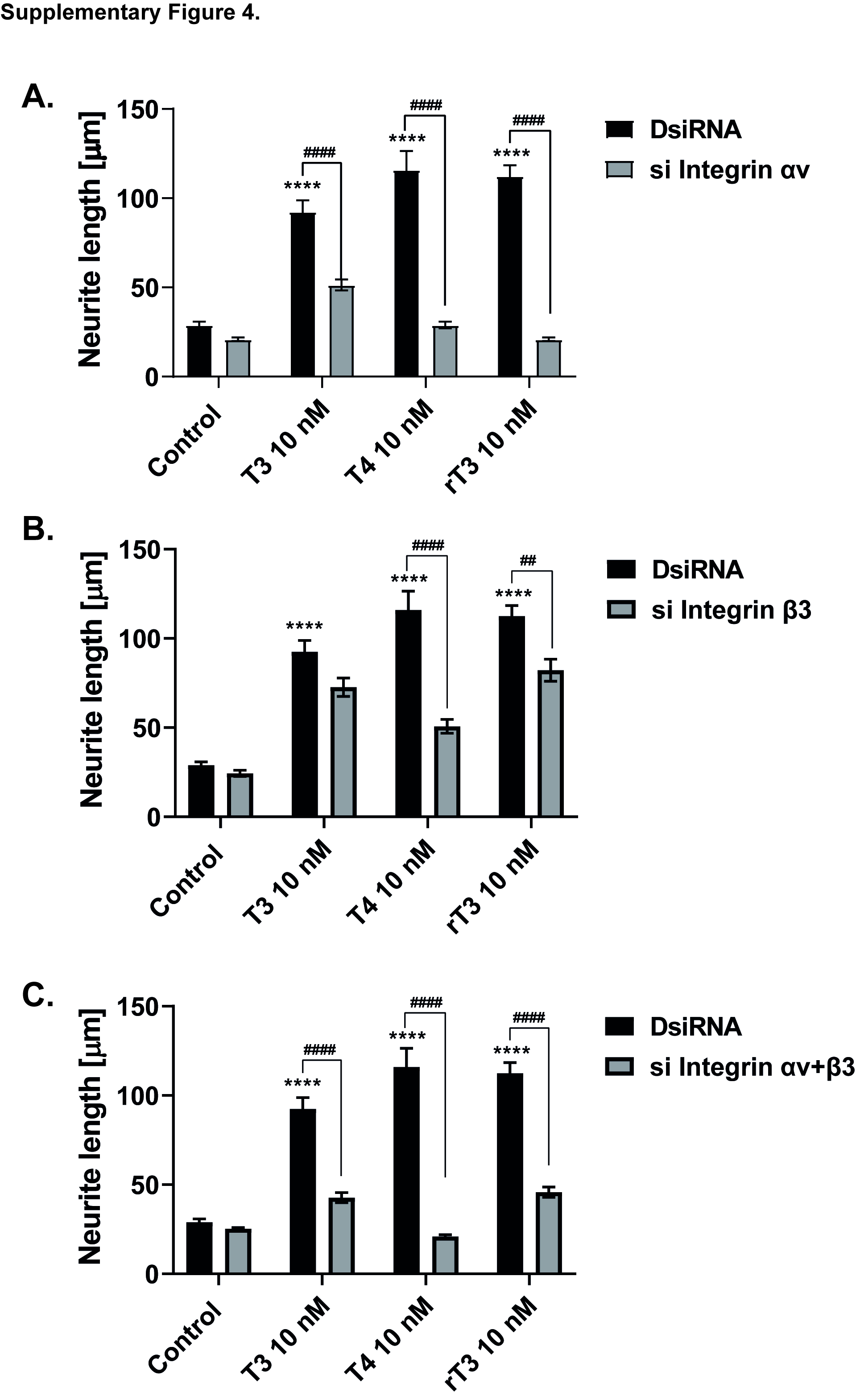

Supplement: Supplementary file 5 [file Image_4.tif]

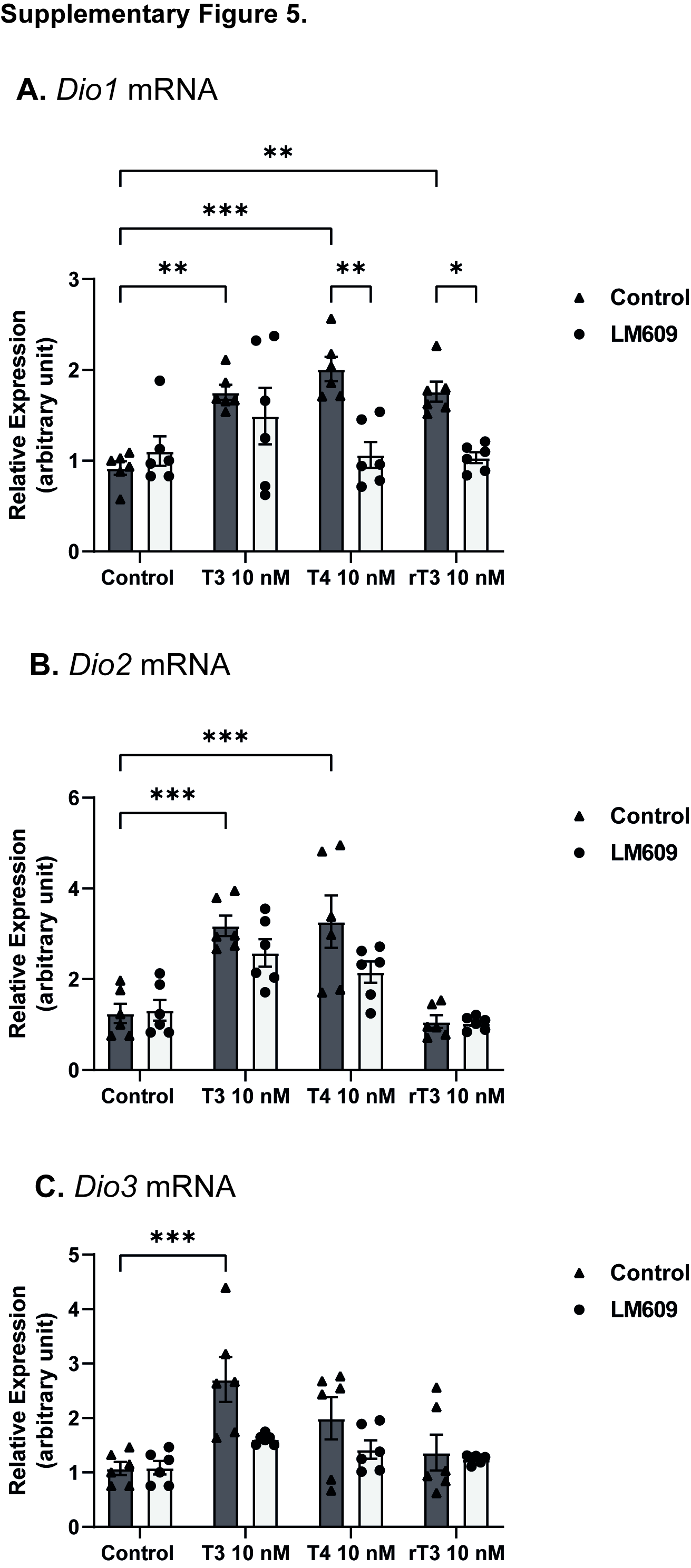

Supplement: Supplementary file 6 [file Image_5.tif]
